# Virtual opioid agonist treatment: Alberta’s virtual opioid dependency program and outcomes

**DOI:** 10.1186/s13722-022-00323-4

**Published:** 2022-07-28

**Authors:** Nathaniel Day, Maureen Wass, Kelly Smith

**Affiliations:** 1 Addiction & Mental Health, Alberta Health Services, Ponoka, AB Canada; 2 Virtual Opioid Dependency Program, P.O. Box 1000, T4J 1R8 Ponoka, AB Canada

**Keywords:** Opioid agonist therapy, Telehealth, Telemedicine, Low barrier, Rural, Virtual

## Abstract

**Background:**

Virtually delivered healthcare (telehealth, telemedicine) has the potential to reduce gaps in access to opioid agonist therapy (OAT). Barriers to accessing OAT such as lack of transportation, in-person induction requirements, employment demands and limited childcare options reduce treatment opportunities for clients. A completely virtual model of care has been developed in Alberta, Canada. This paper introduces the unique virtual clinic model and describes outcomes from that model.

**Methods:**

A retrospective chart review was conducted using datasets within existing electronic health records and databases from Alberta’s Virtual Opioid Dependency Program (VODP). Outcome data were extracted at admission to ongoing care by Case Management within the VODP and at 3, 6 and 12 months for the duration of treatment. Utilization trends over three years were analyzed, including admissions, discharges and active client information. Data regarding clinical outcomes for clients engaged in ongoing care with the VODP were aggregated for analysis over four time periods, including treatment retention rates at 6 and 12 months.

**Results:**

A total of 440 client records were included in the study sample. Descriptive analysis showed rapid growth in utilization over three fiscal years. Despite rapid growth in utilization, median wait days for treatment decreased from 6 to 0 days with the initiation of a Same Day Start service to support low barrier immediate access to treatment. Treatment retention rates for clients in ongoing care were comparable to published reports, with 90% of the study sample remaining in treatment over 6 months, and 58% showing retention over 12 months. Clients reported high levels of satisfaction (90%) and outcomes reflected reductions in drug use and overdose as well as improved social functioning.

**Conclusions:**

The VODP model demonstrated high levels of client satisfaction, rapid growth in utilization and positive preliminary clinical outcomes. Entirely virtual delivery of opioid agonist therapy is a promising option to facilitate access to evidence based treatment for opioid use disorder (OUD) in the context of a fentanyl overdose crisis, particularly for individuals living in rural or underserved areas.

## Background

The misuse of opioids including illicit fentanyl has been escalating in Canada and North America for more than ten years, and negative health effects continue to be reported [1]. In Canada, more than 16,364 apparent opioid-related deaths were documented between January 2016 and March 2020 [2]. Western Canada has been the most significantly impacted region of the country, but numbers have risen in other areas.

Opioid use disorder (OUD) is a chronic health condition that requires long-term supportive treatment. Typical treatment includes pharmacological and psychosocial interventions to reduce drug use while improving psychosocial outcomes [3]. OAT (e.g., methadone or buprenorphine) is a treatment for individuals with OUDs involving the use of opioid agonists (full and partial) in place of higher risk opioids with the goal of maintenance and stabilization. Therapies like these have shown improvements in clients’ psychosocial functioning associated with longer periods in treatment [4]. OAT has been found to be one of the most effective treatments for OUD [5, 6]. Individuals struggling with OUD face many barriers that impede access to care, including stigma, the availability of trained clinicians, and costs associated with treatment [7, 8].

OAT is provided in a number of in-person healthcare settings within Canada [9]. Treatment may be obtained in primary care environments, specialized addiction programs, within in-patient hospital settings, and in federal and provincial correctional facilities [10, 11]. Many programs use a mix of OAT and psychosocial interventions [12, 13]. OAT is delivered in accordance with a variety of jurisdictional regulations across the country. Treatment is typically provided via witnessed dosing at a clinic, or pharmacy, and is tied to strategies to reinforce client participation that include increasing the number of doses that a client is able to take home as they stabilize [9]. Prescribers routinely consider suitability for take-home doses consistent with standards and guidelines for setting expectations regarding take-home doses and witnessed dosing governed by professional colleges. Unfortunately, the need for frequent clinic visits, observed dosing, and limits on take-home doses may also decrease access to OAT for clients who may have competing work demands or need to travel great distances between home and provider.

Some authors have proposed that one strategy to address access as a barrier would be to leverage technology to extend the range of options for psychosocial supports and client check-ins [14]. The use of telehealth to enable consultation, assessment and treatment is an established practice within mental health care and has documented high levels of client and provider satisfaction [15]. It has been suggested that telehealth could reduce both limitations posed by geography and stigma for OAT [16–19].

Telehealth is the delivery of healthcare using synchronous (in real time) and/or asynchronous (via store-and-forward) telecommunications technology. Sometimes referred to as virtual care, telehealth may be defined as “any interaction between patients and/or members of their circle of care, occurring remotely, using any forms of communication or information technologies, with the aim of facilitating or maximizing the quality and effectiveness of patient care” [20]. A systematic review of telemedicine-delivered interventions for substance use disorders (SUDs) identified studies examining the effectiveness of real-time telehealth interventions, including several involving clients with OUDs, noting that most investigations reflected that client satisfaction was consistently high for telemedicine interventions, particularly where access to care was limited [21]. The authors indicated that further studies were needed as there were very few methodologically sound studies in this area. Additional evidence suggests that telehealth, while underused in some settings to treat SUDs, might expand options for treatment for clients in underserved areas [22].

With the advent of the COVID-19 pandemic in early 2020 many jurisdictions in Canada and the United States expanded their use of telehealth to deliver OAT. Regulatory changes in North America were enacted to allow for greater flexibility in the prescribing of medications for the treatment of OUD [23, 24], allowing health care systems to incorporate technologies to support remote care. A number of authors have described their experiences with using telemedicine to initiate buprenorphine treatment in both rural and urban settings [25, 26], suggesting that telemedicine for OAT initiation can reduce or eliminate barriers to treatment, particularly for vulnerable populations such as people being released from incarceration. Wang et al. [25], describing the benefits of telemedicine realized in their OAT clinics in New York State, noted that telemedicine also enables improvements in the referral and engagement of patients needing OAT by using available technology in the community to meet the needs of patients when a crisis arises. Authors in a similar clinic setting in Rhode Island [26] developed a 24/7 telephone service for individuals with OUD to access a buprenorphine prescriber in real time for assessment and OAT initiation as well as an Emergency Department callback protocol to potentially connect patients who have had a recent overdose with care. These authors describe the primary benefits to patients as the ability to engage treatment the instant they become ready to do so without the challenges of clinic hours, transportation or available internet.

In a qualitative study of the experiences of a small group of adults receiving OAT with buprenorphine in a nurse-practitioner facilitated telehealth service, findings supported the use of telehealth for OAT [27]. The three primary themes outlined were “improved access to care”, “isolation”, and “feeling normal on buprenorphine.” Participants related positive experiences with telehealth and described the benefits of online scheduling, reduced travel and individualized care while also acknowledging feelings of isolation, as they tended to have less physical interactions with others in groups and individual sessions than might have been available in an in-person setting.

Scoping reviews have assessed a range of innovations the care of patients with OUD during the pandemic. Krawczyk et al. [28] reviewed treatment services for OUD and harm reduction within various clinical settings over five continents. Innovations aimed at adapting service provision to the pandemic were mostly related to the expansion of telehealth services, with other service modifications pertaining to improving the availability and distribution of medications for patients who were isolating at home. A second scoping review specifically examined telehealth innovations being used to treat patients struggling with OUD with buprenorphine [29]. Telehealth was defined broadly to include virtual visits, telephone, text messaging, other messaging, and mobile applications. Studies reviewed assessed the impact of telehealth on patient satisfaction, treatment retention, access to buprenorphine and adherence. The authors found that the incorporation of telehealth technology was associated with high levels of patient satisfaction, similar treatment retention, and has supported increased access to buprenorphine by reducing geographical and system-wide barriers to care. As the study included articles from 2008 to March 2021 it offered a wide-ranging review of the literature regarding telehealth and OAT, both prior to and during the COVID-19 pandemic. The positive impact of telehealth on the delivery of OAT with buprenorphine was evident across studies and over different modalities.

Limited data exist to document the use of exclusively virtual methodologies to deliver OAT in Canada, particularly in geographic areas with communities situated in rural and remote locations. Previous studies evaluating telemedicine services for the delivery of OAT in Ontario have demonstrated support for the equivalence of telemedicine to in-person care for individuals requiring OAT on measures such as mortality and treatment retention [30, 31], and reflect that it can be challenging for people to access care in rural and northern communities as distances to clinics can be vast and trained clinicians are less available than in urban environments [31]. Eliminating in-person requirements for OAT can potentially expand access to care for people who would otherwise be excluded from evidence-based treatment due to barriers such as in-person check-ins, travel to attend appointments, or observed dosing requirements. This report introduces Alberta’s Virtual Opioid Dependency Program (VODP), a service delivered completely by virtual means with no in-person component of care. The VODP supports individuals from anywhere in Alberta, with the majority of clients referred from rural and regional home communities across Alberta, including those who live and work in remote northern locations.

## The VODP model of care

The VODP leverages existing provincial telehealth infrastructure to deliver an entirely virtual OAT service to clients in almost any location in Alberta, while adhering to best practice guidelines for the use of telemedicine/telehealth to support services for individuals with OUD [23]. The program began in the spring of 2017 as a pilot program to support the provision of OAT in central Alberta, and was expanded in 2018 to accept clients from across Alberta. The team is physically located in Ponoka, Alberta at the Centennial Centre for Mental Health and Brain Injury within the Alberta Health Services (AHS) Addiction and Mental Health portfolio.

Referrals may be made to the VODP using a single toll free number. Clients may self-refer or can be referred from any practitioner or service including agencies such as Corrections, medical detox sites or harm reduction services. Assessment and treatment are provided virtually, in collaboration with existing supports including local to the client laboratories for urine drug screening, community mental health clinics for in-person addiction counseling and pharmacies for supervised dosing. Communication with laboratories and pharmacies is accomplished by telephone and fax, including identification of missed doses by pharmacists and laboratory requisitions for urine drug screens and other tests to support OAT. VODP physicians manage medications and are available for medical consultation, while multidisciplinary teams made up of nurses, social workers, addiction counselors, mental health therapists and other professionals support clients in their recovery journey. Peer support is also available for clients seeking to engage individuals with lived experience.

In 2019 two new service teams were added, including the Same Day (emergency) Start Service and the Transitional Treatment Service. The Same Day Start Service ensures that people who are using street obtained opioids can start treatment the same day they call in, ensuring rapid access to treatment in a moment of crisis, reducing delays in initiation. The Transitional Treatment Service offers client transition support to family physicians, other Opioid Dependency Programs, and primary care in various settings across Alberta. The Transitional Treatment Service provides bridging of prescriptions, connection to local services, and other short-term supports to ensure that no individual experiences gaps in care when transitioning communities or providers.

In 2020 the VODP extended and expanded options for brief supportive addiction counseling and, when needed, referrals to more in-depth therapy to clients while in-program. Addiction Counselors and Mental Health Therapists offer short-term evidence-based therapeutic approaches including goal-oriented methods such as Motivational Interviewing and solution-focused interventions to facilitate resiliency and promote healthy behaviors. For those transitioning back to their home community, referral to local mental health and addiction supports enables continuity of care where appropriate. The VODP also facilitates bridging of OAT care to residential and in-patient addiction and concurrent disorder services to reduce gaps in treatment. Since initiation the VODP has supported clients from more than 240 communities across Alberta.

First client contact involves an initial assessment to determine if urgent same day access to treatment medications is required. If an individual self-refers that assessment occurs via technology during the initial call. When referred from elsewhere VODP contact the client to initiate care. If a client has been using street-obtained opioids, typically fentanyl or analogues, their use and the desire for help is viewed as a “medical emergency”. The client completes an initial intake and is then assessed virtually in the moment by a prescriber. If appropriate the client can be started on medications immediately. Once the client achieves initial stabilization on a treatment medication, a determination is made if the individual has access to appropriate local supports, for example a primary care provider trained and willing to take on OAT care or proximity to an in-person OAT specialty clinic. If there are no appropriate local supports in place, or if other circumstances exist that would preclude a person from participating in local in-person care, then the client is connected with VODP ongoing treatment. Clients supported by VODP ongoing care check in regularly by phone and attend scheduled video visits with prescribers and allied health staff to maintain close monitoring of treatment progress and allow for rapid response to issues as needed.

A client may have more than one admission within a date range, and may engage the VODP multiple times, resulting in more than one admission/discharge cycle. Not all clients admitted into the VODP are retained for ongoing care by the VODP. Wherever possible clients are stabilized and transitioned to local in-person providers. Those who are not are maintained in ongoing virtual care. The proportion of clients retained by VODP Case Management varies from year to year; in 2019/2020, 37% of clients referred to the VODP were retained for ongoing virtual care in Case Management. Clients may be discharged for a variety of reasons, including loss of contact, non-compliance with treatment parameters, transition to an alternative environment for care such as residential treatment, transfer to a local provider, and completion of treatment.

The VODP model was developed to utilize existing AHS telehealth infrastructure to connect a multi­disciplinary team with clients requiring OAT, with all services delivered using telehealth technologies (telephone, text, and videoconferencing). Clients attended video consults at the closest AHS telehealth site to their home community from April 1, 2017 to the end of March, 2020. AHS telehealth endpoints may be found within hospitals, community health centres, and clinics across the province. These endpoints do not have local to the site VODP staff. As of April 1, 2020 video visits were transitioned to AHS approved software-based videoconferencing to comply with safety parameters associated with COVID-19 pandemic protocols, however the current investigation involved only site-based video visits, as it was completed pre-pandemic.

The purpose of this study was to introduce the VODP clinic model, a virtual OAT clinic model that is delivered completely by virtual means for clients living anywhere in Alberta, Canada. Objectives included the description of preliminary evidence of acceptability, treatment retention and outcomes for clients in ongoing care with the VODP, as measured by reported substance use, client reports of overdoses, client ratings of pain and assessment of OUD symptom severity, social functioning and client satisfaction over re-assessment.

## Methods

### Study design

The study design was a retrospective descriptive observational investigation via chart review using a fixed available sample from datasets within existing electronic health records and databases for clients admitted to ongoing care by VODP Case Management between April 1, 2017 and March 31, 2020. This initiative met ethics review requirements as specified by Alberta Health Services.

### Data sources

The dataset was derived from information from two electronic databases: ASIST (Addiction & Mental Health System for Information and Service Tracking) and REDCap [32]. ASIST maintains records of utilization and reports at an aggregate level (admissions, discharges, active clients, length of stay). For clinical outcomes related to the study sample the outcome measurement tool was the ODP Admission Assessment and Re-assessment Project managed using REDCap electronic data capture tools hosted by AHS. This project serves as an ongoing clinical data collection tool to monitor client status at admission to Case Management in the VODP and at 3 months, 6 months, and at 12 months for clients while in treatment. REDCap questionnaires were administered by the most responsible VODP Case Manager or delegate by telephone and responses were entered into REDCap. All clients are encouraged to complete questionnaires but they are not mandatory. Clients may decline to complete individual items, may terminate the assessment session at any time, or refuse to participate in the follow-up assessments entirely.

### Study sample

**Fig. 1:**
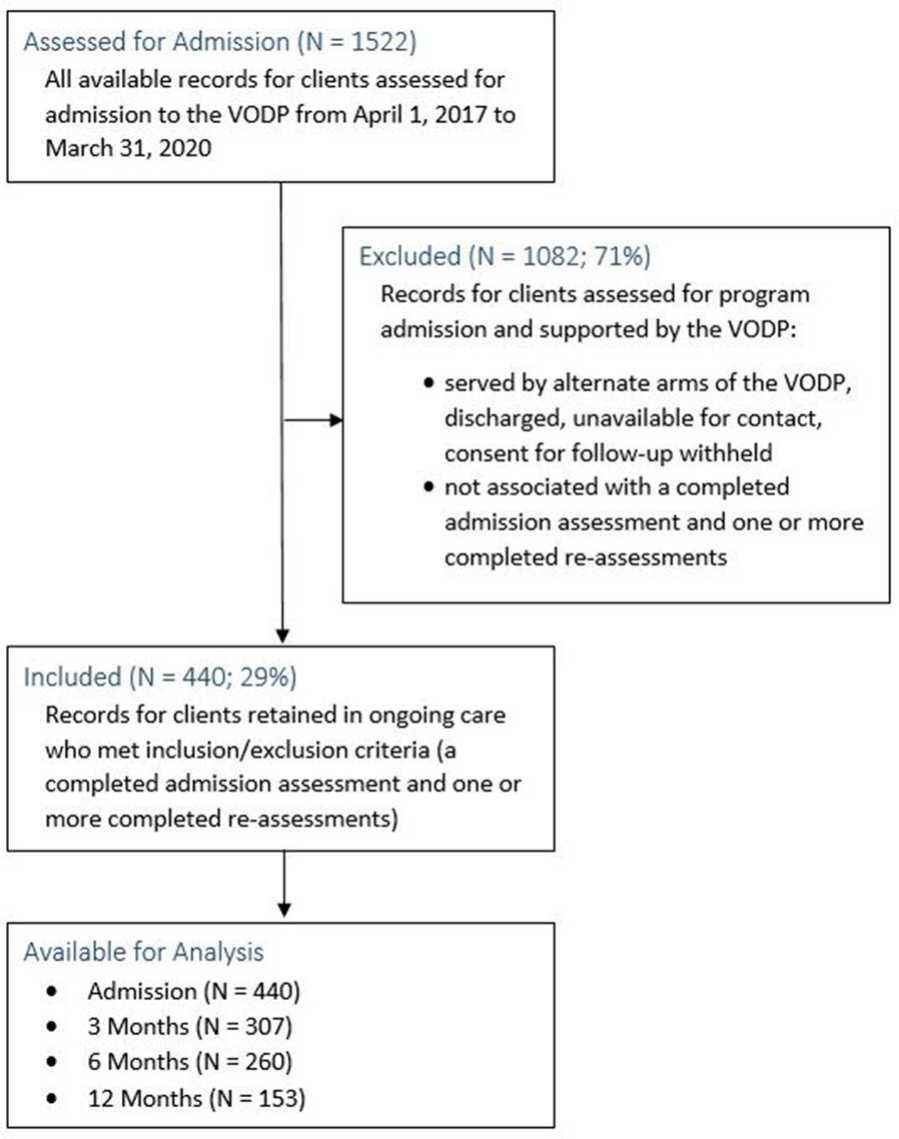
Flow chat outlining steps to create study sample

Clients in ongoing care with the VODP were 18 years of age or older and were living in communities across Alberta. The main study sample was identified by first extracting all available records of REDCap questionnaires for clients admitted to the program between April 1, 2017 and March 31, 2020 (N = 1522). Only clients retained for ongoing care by the VODP were included in the study sample; as many as two thirds were served by alternate arms of the VODP such as the Transitional Treatment Service, the Same Day Start Service, or are discharged. These clients were not followed over time as support through transition can be targeted and brief, and thus were excluded from the study. Client records were included in the sample for analysis if they evidenced a completed admission assessment and at least one complete re-assessment. A total of 440 unique client IDs (identifiers removed) were available for inclusion. The steps used to create the final sample are outlined in Fig. 1.

Clients in the original group who were assessed for admission to the VODP but who did not go on to complete further re-assessments may have done so for various reasons. They may have continued in treatment but declined to participate in follow-up assessments, as clients are not required to participate and there is no change made to their care if they choose to opt out. They may, in alternative situations, have been in the program for up to 3 months and then elected to not continue in treatment. Others may have transitioned to a provider in their local community. Finally, a small number of clients would have been triaged as Harm Reduction, in which case re-assessments are not completed due to contact challenges.

### Measurement

Program characteristics for the VODP over three years were extracted from ASIST. Program characteristics describe all clients admitted to the VODP, including those engaged in ongoing care by VODP ongoing care but not exclusively those individuals, as the VODP offers multiple service arms supporting client engagement in OAT. The number of active clients refers to the number of individual clients who were active with the program during the indicated time period.

Outcome measures related only to clients retained by VODP Case Management in ongoing care. Treatment retention was calculated at 6 months and at 12 months for clients in the study sample. Outcomes were extracted from the ODP Admission Assessment and Re-assessment Project maintained in REDCap on admission and at 3 months, 6 months and 12 months while the client was in treatment. The primary outcome measure was Drug Use, and the questionnaire item was worded, “Are you using this drug?” with binary response options (i.e., yes or no). Drug Use follow-up referred to behaviors during the previous 3 months. Secondary outcomes included the number of categories of substances used by each client within 30 days of assessment, with a score out of 7 calculated with one point added per category (Category 1: Fentanyl/Heroin; Category 2: Other Opioids-Codeine/Tylenol 3, 4; Hydromorphone; Oxycodone; Methadone; Morphine & Other); Category 3: Cocaine/Crack Cocaine; Category 4: Amphetamines; Category 5: Methamphetamines (Crystal Meth); Category 6: Benzodiazepines/Tranquilizers; Category 7: Other Drugs). Of note, when in the pilot stage, the VODP did not provide methadone initiation and as a result methadone was included as one of the substances of interest. To maintain data integrity for this evaluation methadone was retained as a substance of interest despite the fact that methadone was added as a treatment option once initial pilot safety monitoring and expansion were completed.

Additional outcomes included the client's self-reported history of accidental and intentional overdose, with questionnaire questions worded, “Have you accidently overdosed?” and, “Have you intentionally overdosed?” The time frame for follow-up was over the previous three months and response options were binary (i.e., yes or no). Clients' ratings of their level of pain during the 30 days leading up to admission and each re-assessment were also assessed. The variable question was, “Please select one of the following choices to rate your level of pain in the past 30 days: ‘no pain’ is associated with 0, and ‘worst pain possible’ is associated with 10”. The severity of symptoms related to opioid use was also identified as an outcome, derived from diagnostic criteria for Opioid Use Disorder found in the *Diagnostic and Statistical Manual of Mental Disorders* (5th ed.) [33]. This item was scored based on the checklist described in the DSM-5, and the final scale for this item was None (0–1 symptom), Mild (2–3 symptoms), Moderate (4–5 Symptoms), and Severe (6 + symptoms). The final response for this item was scored and recorded by the client’s Case Manager or delegate who administered the interview.

Items from the Brief Treatment Outcome Measure [34, 35] Social Functioning Scale (BTOM-SFS) were embedded in the project, and scores were extracted at admission, 3 months, 6 months and 12 months. The 6 item social functioning scale includes measures of personal and social well-being related to the client’s levels of financial hardship, conflict in relationships with spouses/partners, other relatives and employers/school staff and students, time spent living with person(s) who use drugs and time spent with friends who do not use drugs. Responses related to how often or how much time clients reported that they spent engaged in these behaviors during the previous 3 months. A composite score was calculated for each of six sub-scales for each client based on Likert scale responses between 0 and 3, with lower scores reflecting better social functioning.

Clients' ratings of satisfaction with using telehealth in support of their OAT care on a 10 point scale ranging from 1 (Completely Dissatisfied) to 10 (Completely Satisfied) were identified as outcomes and were available for extraction at 3 months, 6 months, and 12 months within re-assessments, as were client ratings of satisfaction with the program (ranked on an ordinal scale with five response options: Very Helpful, Somewhat Helpful, Undecided, Not So Helpful, Not At All Helpful). Open-ended responses describing what clients found most helpful and least helpful about the VODP were also extracted at 3 months, 6 months, and 12 months at each re-assessment, and were were exported verbatim.

### Statistical methods

Data were analyzed using SPSS (version 25) and Microsoft Excel (2013). Descriptive statistics summarized quantitative responses including measures of central tendency and frequencies across four assessment periods. Descriptive statistics were presented for dichotomous variables (i.e., yes or no), and non-parametric Friedman tests of differences over repeated assessments were performed for ordinal variables (Likert Scale or numerical ratings) and non-normally distributed continous variables. Where variables were ordinal but missing data precluded completion of Friedman tests of differences, descriptive statistics were presented. Responses to open-ended questions were pooled across re-assessments and analyzed using NVivo (version 12, 2019) to allow for coding and organization into themes. A small core group of coding categories were developed a priori, with additional elements emerging from the analysis.

There were two main sources of missing data identified within the study sample. Variations in workload demands on program staff and unavailability or unwillingness of clients to complete assessments in a timely fashion resulted in gaps in data collection leading to missed or incomplete assessments within the series for a number of client IDs. For example, for the 226 admission assessments associated with one re-assessment, 133 (59%) were paired with 3 month re-assessments, 72 (32%) were connected with 6 month re-assessments, and 21 (9%) were associated with 12 month re-assessments. Secondly, within individual assessments, singular items were left uncompleted. Due to the retrospective nature of the study, no explanations were available for the origin of those missing values, and patterns of individual missing responses were assumed to be missing at random as they were distributed across re-assessments. No imputation was used to address missing data in the statistical analysis, and listwise deletion was employed for Friedman tests when variables were ordinal, therefore only responses for client IDs with all four assessments completed were analyzed.

## Results

A total of 1522 client IDs were identified for potential inclusion in the study sample. Of those, 1082 were excluded as they were not retained in ongoing care because they were supported by alternate arms of the VODP, were discharged, were unavailable for contact or withheld consent for follow-up and thus were not associated with a completed admission assessment and one or more completed re-assessments. There were 440 client IDs that met the inclusion criteria and were available for analysis. Those client IDs made up the study sample, and were associated with 440 admission assessments, 307 three month re-assessments, 260 six month re-assessments and 153 twelve month re-assessments as shown in Fig. 1.

The VODP has seen increases in utilization over three years since launch while reducing median wait days for medication initiation from 6 days to zero. The number of unique active clients supported by the program increased from 201 to 2017/2018 to 1225 in 2019/2020. New admissions to the program went from 213 to 1247 for the same three year time period. Median length of stay has fluctuated, initially at 138 days for the first year after launch, rising to 233 days in 2018/2019 and decreasing to 154 days in 2019/2020.

**Table 1:** Study sample client characteristics at admission

Variables	Study sample (*N* = 440)
Age (mean years)	37.7
Age (SD)	11.8
Gender	
Male, *N* (%)	246 (55.9%)
Female, *N* (%)	194 (44.1%)
Education	
< High School, *N* (%)	175 (39.8%)
High School or Equivalent, *N* (%)	140 (31.8%)
Some College No Degree, *N* (%)	95 (21.6%)
Undergraduate Degree, *N* (%)	20 (4.5%)
Graduate Degree, *N* (%)	5 (1.1%)
Missing, *N* (%)	5 (1.1%)
Employment	
Unemployed, *N* (%)	297 (67.5%)
Employed, *N* (%)	110 (25.0%)
Student, *N* (%)	2 (0.5%)
Missing, *N* (%)	31 (7.0%)

Study sample client characteristics at admission were derived from REDCap records and are summarized in Table 1. Average age was 37.7 years (*SD* = 11.8), and clients identified as 44% female and 56% male. The majority of clients were unemployed at admission. Approximately 25% of clients in the sample reported some form of employment, and 50% of clients who reported a change in their source of income in the prior three months (N = 171) described new income from employment (full or part time and casual). The remaining 50% described other changes, including layoff, initiation of income supports, or reliance on savings/pension. Employment data were unavailable for 31 client IDs. Median length of stay was over one year for the study sample, with 90% of the study sample showing treatment retention of over 6 months, and 58% demonstrating retention over 12 months.

### Drug use

Fentanyl/Heroin use was most commonly reported on admission assessment (65% indicated use). There were reductions in the reported use of most substances (see Table 2). Three of the 440 client IDs were missing data for all substances.

**Table 2:** Drug use

Variables	Admission (*N* = 440)	3 Months (*N* = 307)	6 Months (*N* = 260)	12 Months (*N* = 153)
Fentanyl/Heroin, *N* (%)	287 (65%)	43 (14%)	15 (6%)	7 (5%)
Codeine/Tylenol 3/4, *N* (%)	108 (25%)	12 (4%)	6 (2%)	0 (0%)
Hydromorphone, *N* (%)	118 (27%)	5 (2%)	2 (1%)	2 (1%)
Oxycodone, *N* (%)	176 (40%)	9 (3%)	7 (3%)	0 (0%)
Methadone, *N* (%)	88 (20%)	25 (8%)	18 (7%)	18 (12%)
Morphine, *N* (%)	102 (23%)	12 (4%)	1 (0%)	2 (1%)
Other Opioids Used, *N* (%)	53 (12%)	0 (0%)	3 (1%)	0 (0%)
Cocaine/Crack Cocaine, *N* (%)	127 (29%)	27 (9%)	20 (8%)	9 (6%)
Amphetamines, *N* (%)	21 (5%)	7 (2%)	5 (2%)	3 (2%)
Methamphetamines, *N* (%)	107 (24%)	28 (9%)	12 (5%)	6 (4%)
Benzodiazepines/Tranquilizers, *N* (%)	121 (28%)	36 (12%)	25 (10%)	18 (12%)
Other Drug Use, *N* (%)	31 (7%)	19 (6%)	18 (7%)	15 (10%)
Missing, *N (%)*	3 (1%)	3 (1%)	3 (1%)	3 (2%)

### Poly-substance use

The frequency of the use of one or more drug types declined from admission assessment to re-assessment(s), with 90% reporting the use of multiple substances on admission to VODP ongoing care (N = 440, 7 missing), and only 29% of respondents remaining in the VODP at 12 months reported continuing to use one or more types of substances (N = 153, 4 missing). For the 61 cases with complete data at each time point (defined by scores out of 7 calculated for each category of substance used), a Friedman test of differences over repeated assessments rendered a χ2(3) of 82.477 which was significant (p < .001).

### Overdose

Reductions were observed in reported accidental overdoses with similar reductions for intentional overdoses, shown in Table 3.

**Table 3:** Accidental and intentional overdose

Variables	Admission (*N* = 440)	3 Months (*N* = 307)	6 Months (*N* = 260)	12 Months (*N* = 153)
Accidental Overdose, *N* (%)	168 (38%)	29 (10%)	33 (13%)	17 (11%)
Missing, *N* (%)	4 (1%)	4 (1%)	2 (1%)	0 (0%)
Intentional Overdose, *N* (%)	48 (11%)	9 (3%)	16 (6%)	6 (4%)
Missing, *N* (%)	13 (3%)	5 (2%)	1 (0%)	2 (1%)

### Opioid use disorder symptom severity score (DSM 5)

Opioid use symptom severity ratings were extracted and descriptive statistics are shown in Table 4. The frequency of ratings of “severe” symptoms decreased over re-assessments, however less than half (48%) of study sample client IDs demonstrated completed scoring on admission for this variable. As there were significant gaps in data availability for this variable it was determined that there was not enough information for statistical analysis over four time periods.

**Table 4:** DSM 5 opioid use disorder checklist severity rating

Variables	Admission (N = 440)	3 Months (N = 307)	6 Months (N = 260)	12 Months (N = 153)
None (0–1 symptoms), *N* (%)	34 (8%)	57 (19%)	70 (27%)	53 (35%)
Mild (2–3 symptoms), *N* (%)	9 (2%)	20 (7%)	23 (9%)	16 (10%)
Moderate (4–5 symptoms), *N* (%)	14 (3%)	5 (2%)	2 (1%)	0 (0%)
Severe (6 + symptoms), *N* (%)	154 (35%)	10 (3%)	4 (2%)	0 (0%)
Missing, *N* (%)	229 (52%)	215 (70%)	161 (62%)	84 (55%)

### Management of pain

Scores reflecting “no pain” (0) represented 41% of respondents on admission assessment and just over 50% of respondents averaged over all available re-assessments, suggesting that client ratings of their level of pain in the previous 30 days remained relatively low over time in treatment. Median self-reported pain ratings on admission and across re-assessments are outlined in Table 5, reflecting little change in overall client ratings of levels of pain across re-assessments. For the 30 cases with complete data at each time point, a Friedman test of differences over repeated assessments showed a χ^2^(3) of 6.019 (p = .111), which was not significant.

**Table 5:** Pain ratings and BTOM-SFS composite scores

Variables	Admission (*N* = 440)	3 months (*N* = 307)	6 months (*N* = 260)	12 months (*N* = 153)
Median pain rating	3.0	0.0	0.0	0.0
Interquartile range	6.0	5.0	5.75	6.0
Missing, *N* (%)	99 (23%)	90 (29%)	54 (21%)	11 (7%)
Mean BTOM-SFS composite score	4.9	3.2	3.0	2.5
Standard deviation	4.0	3.0	2.9	2.5
Missing, *N* (%)	1 (0%)	3 (1%)	3 (1%)	1 (1%)

### Social functioning

Summary statistics for BTOM-SFS composite scores across assessment periods are shown in Table 5. Patterns of mean BTOM-SFS scores reflected client self-reports of improvements in social functioning over repeated assessments. For the 65 cases with complete data at each time point, a Friedman test of differences over repeated assessments offered a statistically significant result of χ^2^(3) = 22.952 at p < .001.

### Client satisfaction

Satisfaction data were available for analysis over all re-assessments collected at 3 months (N = 307), 6 months (N = 260), and 12 months (N = 153) for clients in treatment. Answers to all satisfaction items were combined over all three re-assessments to give a sample of 720 potential responses. On a ten point rating scale of satisfaction with telehealth, clients reflected high levels of satisfaction, with a median rating of 10 (interquartile range = 1). On a Likert scale item querying how helpful clients found the program, 90% of respondents (N = 720; 5 missing) indicating that they found the program to have been very helpful, and under 1% of respondents rated the VODP as having been not helpful.

On open-ended questions regarding what clients found most helpful and least helpful about the program, components described most frequently as helpful by respondents (N = 720; 8 missing) were staff support and communication (39%) as well as program accessibility (29%). Access to medications for symptoms and pain was also described as helpful by respondents (25%), as was recovery from addiction to re-establish a healthy lifestyle (20%). Aspects of the VODP described most frequently as least helpful by respondents (N = 720; 20 missing) included pharmacy issues related to witnessed dosing and daily pickups (11%), along with challenges related to attending telehealth appointments (8%), such as having to travel to the telehealth site or needing to take time off work. The majority of respondents indicated no concerns or recommendations regarding improvements to the program (61%). Minor concerns centered around various medication issues (bad taste or side effects) were also reported (4%). Other aspects of the program were noted as not having been helpful, such as having to present for lab tests, medication coverage challenges and concerns regarding insufficient pain management, however each of these were described in less than 2% of responses.

## Discussion

This study introduced the Virtual Opioid Dependency Program, a virtual service delivering OAT to clients anywhere in Alberta. Demonstrating steep increases in utilization over 3 years, the program enables access to OAT for clients regardless of where they live or what their circumstances are. In addition to steady increases in the number of admissions and active clients over three years of operation, discharges also rose significantly over time. This was in part due to increasing uptake of the Transition Treatment Service, bridging OAT to community providers where appropriate and resulting in shorter lengths of stay for the program overall. Clients may also return multiple times before engaging in ongoing care with Case Management, which can result in shorter episodes of care. Finally, as utilization increased, discharges have correspondingly increased.

This investigation assessed retention rates for clients engaged in ongoing OAT with the VOPD. In recent studies evaluating telehealth buprenorphine treatment programs for OUDs on several outcome measures including retention rates [36, 37], results of one evaluation revealed that the retention rate for clients who stayed more than 365 days was 41.7% for the telepsychiatry group and 35.5% for the face-to-face group [36]. Weintraub et al. [37] found that clients treated with buprenorphine using telemedicine had good retention after 3 months (57%). These studies were similar to the current investigation in that they retrospectively analyzed data from programs providing medication for the treatment of opioid use disorder to rural populations in the United States. The former investigation assessed the differences between in-person and telehealth group-based OAT regarding time to achieve abstinence and treatment retention. The sample size was small (N = 100) but the study design was strengthened by the addition of the in-person comparison group in the evaluation of treatment outcomes. Results showed good retention beyond one year for the telehealth group (41.7%), similar to the current investigation. In the latter study, investigators conducted a chart review to examine continued use of opioids and treatment retention rates for individuals engaged in buprenorphine treatment using telemedicine at a treatment centre in rural Maryland. The sample size was small but acceptable (N = 177), with no comparison group. Results of a larger follow-up investigation confirmed that treatment retention for the telemedicine group remained at 50% [38]. A systematic review of retention rates for in-person OAT reported median retention rates across observational studies at about 57% at 12 months [39]. And in a cohort study completed in Ontario, Canada [30], results indicated that retention in treatment for telemedicine was better than in-person, with the telemedicine group showing 50% retention at one year compared to 39% retention for the face-to-face group. Retention rates from the present investigation were closely aligned with those found for telemedicine.

The findings from the current study suggest that ongoing OAT care delivered only by virtual means may support reductions in overall drug use while in treatment, along with a decline in reported overdoses experienced while in treatment. Reductions in poly-drug use were also observed, which was consistent with other reports of trends in decreasing multi-substance use after engagement in OAT for some substances [40], and significant in light of studies indicating that poly-drug use can reduce the benefits of drug treatment and increase relapse risk [41]. Investigators in Australia reported that reductions in heroin use were associated with a decline in use of cocaine, amphetamine, cannabis, benzodiazepines and other opioids [42]. These findings were consistent with those of a cross-sectional longitudinal analysis of the co-use of opioids with other drugs to uncover possible associations between co-use and receiving OAT in a sample derived from a large administrative database in Minneapolis [43]. Results showed that poly-drug use of other substances and opioids was associated with significantly lower rates of receiving OAT. A second cross-sectional study sought to define factors associated with positive urine drug screen results for non-prescribed drugs for patients taking buprenorphine over several years [44]. Results reflected that almost half of patients (47.58%) tested positive for non-prescribed substances, and that positive tests for buprenorphine were associated with lower positivity for all other drugs with the exception of gabapentin. While these cross-sectional studies are significantly larger and more statistically robust than our investigation, our observation of reductions in poly-drug use in clients receiving OAT is consistent with previous investigations [40–44]. Reductions in drug use were observed across substance categories and overall, corresponding to previous research findings.

The outcomes of this evaluation suggested positive changes in client reports of social functioning from admission to re-assessments over time. These results align with previous reports of improvements in social functioning for individuals struggling with opioid use disorder who were engaged in in-person treatment with medication for opioid use disorder [45]. While the current study used the BTOM, other authors have selected measures such as the Addiction Severity Index (ASI) [46], making direct comparisons difficult.

OAT has been identified as potentially helpful for providing relief from pain [47]. While the current investigation did not demonstrate changes in subjective pain ratings over repeated assessments, other authors have suggested that improvements in pain ratings while engaged in OAT may be related to the type of drug being used before induction, as well as the amount of opioid being taken before treatment commenced [48]. Other potential influences that may have led to a lack of reduction in client reported pain in the current investigation may be related to clinical differences in the population of clients evaluated, as these clients were not specifically selected as chronic pain sufferers, and could be engaged in care with the VODP because they have an OUD with acute or chronic pain, have an OUD without acute or chronic pain or present with issues just related to pain. It is also possible that the observational study design did not allow for an adequate investigation of the impact of OAT on client ratings of pain, as pain was only one of several outcomes under investigation and subject to external influences not controlled for in this design. Pain ratings were low in the study sample overall, and this may have been the most likely reason why pain ratings did not change. Client ratings of pain did not appear to worsen despite many individuals transitioning from full agonist opioids to a partial agonist opioid.

This report is, to our knowledge, one of the first to evaluate the outcomes and acceptability of an exclusively virtual OAT treatment program serving a geographically diverse and largely rural population in Canada. It is important to consider the potential for response biases such as recall and social desireability biases to impact results as variables pertained to socially stigmatizing behaviors such as substance use and overdose and responses were self-reported. Self-report has been reviewed in other investigations of substance use and has been shown to be a valid and reliable representation of drug use [49–51], however social desireability bias may be more likely to be observed in clinical data than in research interviews, since clients may expect that there could be clinical consequences based on what they report. The investigation used existing data pertaining to interviews where clients were asked to respond to questions administered by their most responsible Case Managers or delegates, and no verification was available for the data when gathered. This may have resulted in interviewer bias as each Case Manager would have had prior knowledge of the health status of the client that might have influenced the results.

The design lacked a comparison group, limiting the generalizeability of the results, and the sample size, while selected to include the maximum number of client IDs available was still relatively small for an observational study, so no direct conclusions may be arrived at. As the study sample was derived from a larger sample of individuals who, for a variety of reasons, did not complete a re-assessment at some point during their ongoing care, this also limits the generalizeability of the results. It is important to acknowledge that because clients in the study sample were individuals who remained in treatment and were agreeable to completing assessments they may have also had more positive outcomes. We cannot rule out that the results observed were not due to other influences or interventions occurring outside of OAT. Because of the nature of the retrospective observational design, missing data resulted in further limitation to the already cautious interpretation of results. Finally there could also have been potential bias due to loss to follow-up, as those clients lost to follow-up may have had poorer treatment outcomes, leading to an underestimate of substance use, overdose and other harms in the re-assessment period.

The results obtained were consistent with those documented in previous research pertaining to telehealth/telemedicine delivery of OAT, however it is difficult to compare our results directly to other investigations as each study described a unique set of outcome measures delivered in specific clinical contexts. Finally, non-parametric tests may have less power than corresponding parametric tests as they are distribution-free, however they are the appropriate choice when data are categorical and not normally distributed, as was the case in this investigation. Strengths of the current study related to the single clinic data source with relatively uniform protocols for follow-up and the same (albeit growing) group of physicians and multidisciplinary team members delivering care.

## Conclusions

The objective of the present study was to describe the development and preliminary outcomes of the VODP, as it is an innovative clinic model of OAT care delivered completely by virtual means (videoconference, telephone, and text) facilitating access to care to individuals in Alberta, Canada. While the results were encouraging, the study was descriptive and retrospective and as such must be interpreted cautiously. Further studies using a prospective randomized controlled design are needed to determine the effectiveness of a virtual treatment model for OAT. As an observational study, no conclusions may be drawn regarding treatment effectiveness, as other influences may have led to the pattern of responses observed.

Clients of the program reported satisfaction and positive outcomes while in treatment. The VODP supports continuity of OAT care for clients to maintain employment and enables improvements in overall lifestyle and healthy behaviors. The VODP model of virtual OAT care offers avenues for growth and expansion supported by new mobile technologies and software-based videoconference platforms to reduce barriers to care for individuals in diverse living situations and those not easily served by traditional clinics. Virtual models of assessment and treatment with OAT offer an opportunity to improve client access while maintaining meaningful quality outcomes in a changing healthcare landscape.

## Data Availability

The datasets used and/or analyzed during the current study are available from the corresponding author on reasonable request.

## Abbreviations

ASIST: Addictions & Mental Health System for Information and Service Tracking
BTOM-SFS: Brief Treatment Outcome Measure-Social Functioning Scale
CADTH: Canadian Agency for Drugs and Technologies in Health
VODP: Virtual Opioid Dependency Program
OAT: Opioid Agonist Therapy
ODP: Opioid Dependency Program
OUD: Opioid Use Disorder
REDCAP: Research Electronic Data Capture
SUD: Substance Use Disorder
SPSS: Statistical Package for the Social Sciences
